# Optimal competition strategy analysis of China Railway Express based on evolutionary game theory

**DOI:** 10.1371/journal.pone.0256326

**Published:** 2022-03-18

**Authors:** Fenling Feng, Xiaojie Li, Junkai Liang, Yang Liu, Chengguang Liu

**Affiliations:** College of Transportation Engineering, Central South University, Changsha, Hunan, China; Institute for Advanced Sustainability Studies, GERMANY

## Abstract

In the context of the Belt and Road Initiative, China Railway Express achieved rapid development. Determining how to achieve effective marking and determining how to adopt the optimal competitive strategy are the main challenges for CR Express operators. By choosing long-distance transportation as the research object, this research established a competitive model between CR Express and maritime transportation based on game theory. First, we determined the participants of this competition. By dividing common goods into the categories of high-value and high-time-sensitiveness, high-value and low-time-sensitiveness, low-value and high-time-sensitiveness, and low-value and low-time-sensitiveness, the model was used to simulate four situations and to obtain optimal strategies for operators. For CR Express, it was always effective to adopt a service optimizing strategy to improve the service level and earn market share. For maritime transportation, this strategy was effective only for high-value and low-time-sensitiveness goods as well as low value and low-time-sensitiveness goods. Therefore, transportation service suppliers should make competitive strategies based on different good types, and it is effective to use differentiation strategies to earn market share and improve competitiveness only for suitable types of goods.

## Introduction

In 2013, Jinping Xi, the president of China, proposed the Belt and Road (B&R) Initiative, consisting of the Silk Road Economic Belt and the Maritime Silk Road, which attracted much attention in international society. In 2015, several ministers and commissions jointly issued the “Visions and Actions on Jointly Building Silk Road Economic Belt and 21st Century Maritime Silk Road”, which first proposed the route planning of the Belt and Road Initiative from the country perspective. Among this, trade development is one of the emphases. By January 2021, China had signed 205 cooperation documents on “the Belt and Road Initiative” with 171 countries and international organizations. “The Belt and Road Initiative” has made important contributions to promoting a new type of economic globalization. As one of the important transportation modes, railway interconnection is of significance. China Railway Express, referring to express container trains from China to Europe, with the initial purpose of enhancing the transportation ability of inland cities, has achieved rapid development and obtained great achievements. The number of CR Express increased rapidly from 17 in 2011 to 8,225 in 2019, an increase of 482.82 times. As of the end of 2019, the cumulative number of trains reached 21,162. It has effectively promoted economic and trade exchanges among countries along the B&R routes.

As a traditional transportation mode, maritime transportation maintains its prime market position due to its low freight rate and comparatively stable service level. To earn market share and achieve coordinated development among China and other related countries, the government provides subsidies to CR Express to face market competition and achieve sustainable development. For long-distance transportation from China to Europe, CR Express and maritime transportation are the main competitors in the transportation market. Owing to the subsidies from the government, the price of CR Express could be equal to or even lower than that of maritime transportation, which caused some of the marine transportation demands to transfer to CR Express and resulted in the increase of the traffic volume of CR Express. However, the increase based on the lower price of CR Express was not sustainable. Once the subsidies gradually decreased, the freight of CR Express would return to a stable price based on market adjustment. This means that the advantages of CR Express that were based on low freight would not exist anymore, and the market competition between both sides would be more intense. Therefore, the strategy of low freight for CR Express to compete with maritime transportation was not sustainable and the transportation demands could not be maintained.

Therefore, in the future development of CR Express, the operator of CR Express must consider how to compete with maritime transportation, how to win market share and other issues. Both the government and the operator of CR Express need to take other the optimal strategy to achieve the sustainable development of CR Express. The aim of this research is to provide operating thoughts for CR Express operators on what optimal competitive strategies there are for the operators to earn market share and achieve sustainable development. Based on game theory, choosing maritime transportation as the main competitors in long-distance transportation, we established a game model to simulate competitions between CR Express and maritime transportation base on differentiation strategy. Common goods were divided into four types, including high-value and high-time-sensitiveness, high-value and low-time-sensitiveness, low-value and high-time-sensitiveness, and low-value and low-time-sensitiveness goods. This research achieved heterogeneous competition simulation and optimal competitive strategies were obtained for both CR Express and maritime transportation operators, which revealed operating thoughts for the different transportation market.

This paper is further organized as follows: Section 2 presents a literature review. Section 3 studies the participants and strategy sets of the game, then builds a evolutionary game model. Section 4 carries out the market segmentation of China-Europe cargo, classifies the railway transportation categories. Section 5 selects CR Express (Chongqing-Duisburg) as a case study the competition between maritime shipping and CR Express. At last, Section 6 concludes the paper.

## Literature review

Since the first CR Express traveled from Chongqing to Duisburg in 2011, CR Express has fascinated many domestic researchers in China. In terms of development countermeasures, the researchers mainly put forward the problems existing in the operation of the CR Express by studying the development status of its operations. Zhao and Guo studied the influence of CR Express on international trade by building a gravity model [1], and they proposed the development plan of CR Express in terms of national cooperation, the logistics level, and competitiveness. By analyzing the problems existing in the operation of CR Express, Wang et al. [2] put forward relevant suggestions based on the aspects of the logistics network, source organization, and subsidy exit mechanism. After studying the waybill and related procedures of CR Express, Dai [3] believed that the waybill of CR Express had some problems, such as difficulty in filling in, difficulty in claiming compensation, and difficulty in settlement. By studying the current situation of the waybill of CR Express, Sun [4] put forward the method of using the multimodal transport bill of lading in the operation of CR Express. Fu et al.took seven China-Europe Express Lines as the research object [5], established an evaluation index system based on multiple aspects, compared and analyzed the value and cost of different routes, screened out the best plan, and put forward relevant countermeasures based on this to improve the economy of China-Europe trains. Li analyzed the current situation and existing problems of China-Europe express trains and proposed optimization countermeasures for China-Europe trains under the "One Belt One Road" strategy [6]. Jang and Fan performed an analysis and proposed that there were problems in the development of China-Europe freight trains [7], such as inconsistent standards, low return traffic, unguaranteed running time, low outbound full load rate, and disorderly competition. Then they put forward specific countermeasures for these problems. Cui and Wu analyzed the development status and existing problems of China-Europe railway transportation corridors [8], and they put forward countermeasures to improve the service quality of China-Europe railway trains using Asia-Europe transportation corridors.

With the deepening of the reform and the marketization of railway freight transportation, railway marketing has become an important means to improve the competitiveness of railway freight transportation, and research on railway freight transportation marketing has become the focus of scholars. In terms of the aspect of the marketing of CR Express, it mainly includes the related operation strategy research, performance evaluation, target freight source selection, and integration mode research. From the perspective of supply chain construction, Wu [9] analyzed the marketing strategy selection of CR Express operating enterprises. Xing [10] analyzed the marketing status and existing problems of the Hao International Logistics Company through the PEST method and proposed corresponding marketing strategies based on STP theory.

Because of the heterogeneity of technical and economic characteristics among various modes of transportation, different modes of transportation have different advantages and application scopes, and the fields with competitive advantages are also different. Under the traditional competition analysis of transportation methods, the impact of cargo differences on the competition of different transportation methods is often not considered. The sensitivity of the cargo owner to the price of the goods is positively correlated with the degree of difference of the goods themselves. Therefore, for the transportation company, it is necessary to formulate a competitive strategy that focuses on the differentiation of the goods. Based on this analysis, research on market competition strategies for CR Express and maritime transportation should conduct market segmentation research on the Chinese and European freight markets. Market segmentation is the key link of marketing. Market segmentation was first proposed by Smith [11], and it has attracted the attention and research of a large number of experts, scholars, and practitioners. Duan et al. divided the market segment by the difference in the selection preference of railway freight customers based on the LC-HB model [12]. Based on the improved K-means clustering method, Deng et al. chose the recent delivery capacity, delivery frequency, freight income contribution rate, and forecast value of delivery tendency as a classified index and carried out customer segmentation [13]. With the aim of minimizing the total operating cost, Wang et al. constructed the optimization operational model of a railway transportation product operating based on the idea of product differentiation [14]. Li [15] aimed at railway bulk cargo transportation, conducted market segmentation based on market factors such as transportation distance and time, and constructed a product system for bulk cargo transportation. Zhang [16] proposed a product development strategy based on a railway dynamic logistics alliance. Shuai et al. [17] used a BCG matrix to classify railway express freight products into dog products, cash-cow products, and star products, and put forward product marketing strategies.

The basic idea of game theory is that the schemes adopted by each player will affect each other, and the gains of each player after the game will not only depend on their own strategies, but also be affected by the strategies adopted by other players [18]. Nash equilibrium is the core concept of game theory, which can also be called the solution of a game. The significance of the Nash equilibrium solution is that once one player in the game adopts an equilibrium strategy, the other players must also use an equilibrium strategy to gain better benefits.

An evolutionary game is the application of biological evolutionary thought in economics. An evolutionary game is established under the bounded rationality of participants. Each participant is affected by the decisions of other participants, and each participant finally reaches a stable equilibrium state through a dynamic selection mechanism [19, 20]. Studies on evolutionary games have mostly focused on the analysis of stable strategy ESS [21–23], the individual adaptive learning mechanism and dynamic regulation of replication [24–26], evolutionary cooperation under mutation conditions [27–29], etc. Wu et al. [30] used an evolutionary game to analyze the evolutionary equilibrium and stability of a horizontally differentiated product and studied the complexity of its evolution. Duan [31] studied the stable strategy of the internal competition of port groups by using the port competing model for single population evolution based on the Bertrand model.

In general, the development time of CR Express is relatively short, and the research on CR Express focuses on the problems existing in its own development and the future development planning. At present, most of the goods between China and Europe are transported by sea. In the future, CR Express still has great development potential, and maritime shipping will be the main competitor of CR Express. However, there is little research on the market segmentation and competition between CR Express and sea transportation, and there is also a lack of dynamic competitive strategy analysis between them. Game theory has been widely used in the field of transportation, but there have been few studies on the application of evolutionary games to the marketing of railway goods. Based on these analyses, this paper applies evolutionary game theory model to the study of the competition between CR Express and maritime market, and analyzes the competition strategies of the two in different market environments.

The aim of this study was to achieve the stable development of CR Express and to solve the current problems such as strongly depending on subsidies, vague market positioning, and future development strategy, based on marketing competing strategies. In this study, according to the different characteristics of different freight, we used the theory of quantification III to implement the China-Europe market segmentation. To achieve the sustainable development of China Resources Express, based on the evolutionary game theory, sea transportation is regarded as a competitor of CR Express, and we study the strategy of stable competition between CR Express and shipping under subsidized and non-subsidy conditions, and analyze the process and results of the evolutionary game. The results of this study can provide a reference for the selection of competitive strategies between CR Express and maritime transportation, and can provide operational ideas for CR Express operators and promote the stable development of CR Express.

## Methods

The main idea of game theory is that the actions (strategies) taken by the rational or limited rational participants will affect each other. In addition, the gain and loss results caused by conflict or cooperation between different parties will not only depend on the behaviors taken by themselves but also be affected by the behaviors of others, so the gain of the game participants is a function of their behavior portfolio. Therefore, the first step of building the game model was to study the participants and strategy sets of the game. The body of the model was then constructed.

### Participants

**Fig 1:**
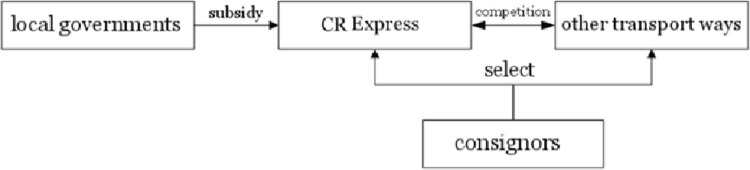
Relationships between market participants.

The game participants were decision makers or the rule designer of the game. As shown in Fig 1, the main participants of the transportation market were transport service providers (carriers), freight service demanders (consignors), and local governments.

During the game process, the consignors chose carriers based on the principle of maximizing utility. The consignors were recipients of the strategies providing by carriers, the local governments aimed to support the development of CR Express and promote local economic development, and the games between carriers were the body of the market competition.

This research focused on the competition strategies with the aim of maximizing market profits and determining solutions to improve competitiveness.

According to the market research results, there were different main competitors for CR Express for different transport distances. For long distances, the main competitor was sea transportation; for short distances, the main competitor was road transportation.

The carriers made different competition and pricing strategies based on the cost of providing service. The cost consisted of human, material, and financial resources required in transportation. In addition, the cost could be divided into the fixed cost and the variable cost. The fixed cost referred to the cost that would not change with the traffic volume during a period of time, which included the operating cost, financial cost, depreciation cost, and amortization of intangible assets [32]. The variable cost referred to the cost that varied with the amount of traffic.

For convenience, in this research, only the fixed cost was considered. We defined *Ci* as the unit transportation cost, where *i* = 1, 2, 3, referring to CR Express, sea transportation, and road transportation, respectively.

#### (1) CR express (long distance)

**Fig 2:**
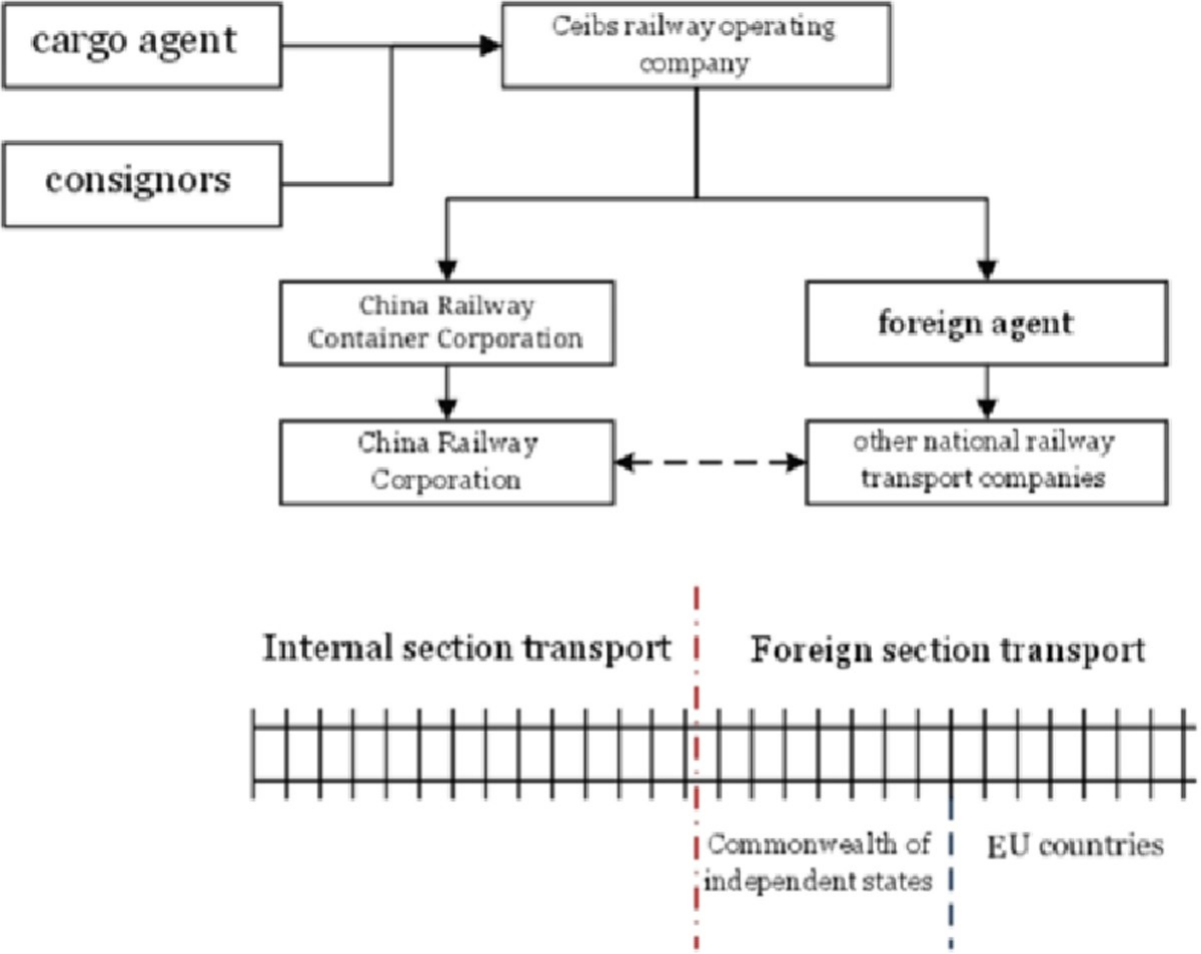
Major participants in the China Railway Express.

CR Express is an international railway through transport that is jointly completed by several national railways from different countries. The participants included consignors, freight forwarders, CR Express operators, the China Railway Container Corporation, and other national railway transport companies. The relationships between the participants are shown in Fig 2 below.

The consignors directly cosigned freight to CR Express operators directly or through freight forwarders. For consignors, the CR Express operators were direct carriers. After the acceptance of freight, the operating companies conducted domestic transportation and outside parts separately. For domestic transportation, the CR Express operators commissioned the China Railway Container Transport Company (CRCTC), and the CRCTC commissioned China Railway (CR) again to conduct transport. For overseas transport, the CR Express operators could only commission foreign forwarders who would choose appropriate transport companies to carry out transportation. For international railway transportation, negotiation among CR and foreign transport companies was necessary.

During this process, although the CRC and the foreign railway enterprises were the actual carriers of CR Express, they only completed the transportation missions of their own stages, and the CRCTC and foreign agents only completed agency tasks in their respective stages. CR Express participated in the cargo supply organization. CR Express was responsible for the safety of goods, facing the test of the cargo owners, and competing with carriers of other modes transportation. In the analysis of the market competition of CR Express, CR Express was selected as the research object.

Taking the 40-foot container transportation as an example, the unit transportation cost of a China-Europe railway carrier was $c_{1}^{op}$.


$$c_{1}^{op}=c_{1}^{in}∙d_{1}^{in}+c_{1}^{out}∙d_{1}^{out}+c_{1}^{ex}$$ (1)

$c_{1}^{in}$——Domestic section of 40 feet container freight rate, in US dollars per box per kilometer

$d_{1}^{in}$——Domestic transport distance, in kilometers

$c_{1}^{out}$——Foreign section freight rate, US dollars per box per kilometer

$d_{1}^{out}$——Transportation distance abroad, in kilometers

$c_{1}^{ex}$——The sum of port agent fees and combined transportation service fees, in dollars

#### (2) Sea transportation

The variable cost of shipping included two parts, which were the cost of parking in the port and the cost of sailing. The detailed composition is shown in Table 1 below.

**Table 1:** Shipping variable cost composition.

Cost composition		Cost division
Fuel cost	This was related to the fuel storage and container usage, including the heavy fuel charge, and light fuel charge.	
Port dues	Ship related	Tonnage tax, berthing fee, anchorage fee, wharf fee, pilot fee, towing fee, cable release fee, quarantine fee, customs inspection fee, agency fee, lighthouse fee, etc.
	Goods related	Tallying fee, switching fee, waiting fee, trimming fee, etc.

### Market game strategy set

Strategies are action rules for all participants under the given environments and known information. Usually, y_i_ represent the strategy of participant i; and the set of the strategy is the sum of all selectable actions or means, which is normally represented as Yi = {y_1_, y_2_, ⋯, y_n_}. To establish a competitive game model between different transportation methods, it was necessary to clarify the strategy set *Y*_*i*_ of each participant.

The strategy set of participants was the sum of the competitive strategies of the participants. In marketing, competitive strategies mainly include three categories, which are the price strategy, cost strategy, and differentiation strategy.

For a transportation service, it was more difficult to change the transportation costs in a short period of time. In addition, the differentiation strategies such as optimizing the service quality and providing logistics services could improve the convenience and flexibility for the consignors and increase market share. Therefore, for operators, the applicable strategies were price strategies and product differentiation strategies.

In the analysis described below, we focused on the strategy choice for CR Express during the long-term repeated game through the evolutionary game model.

### Evolutionary game model

It was assumed that in the whole transportation market, there were only two options, which were CR Express and shipping, referred to as TR1 and TR2, and their strategy space was {using service optimization strategy, no service optimization Strategy}.

Between locations A and B, there were *i* different kinds of transport modes (*i* = 1,2), which indicated railway and maritime transportation, respectively. Assuming that the 40 ft standard container could serve for all of those transportation modes, and the transportation time for the transportation mode *i* was *Ti* (in days), the transportation fee was *τi* (Yuan/FEU). To simplify the analysis, it was assumed that the original value was *Pm*, and the value reduce rate of the goods was constant because the constant devaluation rate and time-varying devaluation rate were the laws of the goods value, which would not affect the transportation choice behavior of the consignor. The value function satisfied the formula:

$$P(t)=P_{m}e^{-\omega t}.$$ (2)

After a time period of *Ti*, the value loss of goods *ΔP* was

$$\Delta P=P(0)-P(T_{i})=P_{m}-P_{m}e^{-\omega T_{i}}=P_{m}(1-e^{-\omega T_{i}}).$$ (3)

Since the consignors had a certain transportation time limit *Tu*, applying faster transportation modes would produce extra profit [33], which was

$$B^{p}=P(T_{i})-P(T_{u})=P_{m}e^{-\omega T_{i}}-P_{m}e^{-\omega T_{u}}.$$ (4)

The parameters related to consumers were the following:

*P*_*m*_: Original value of goods

*ω*: Value reduce rate, constant

*T*_*i*_: The transportation time of the transportation mode *i*

*T*_*u*_: Expected transportation time

Once the goods could not be delivered within the expected transportation time, the value loss of the goods was higher, which caused a penalty cost. The penalty cost consisted of the value loss of goods, the profit loss caused by being out of stock and additional operating costs, etc [34]. For convenience, the penalty cost was regarded as the value loss standard. Therefore, we introduced the penalty coefficient γ. When *T*≤*Tu*, there was no penalty cost, so *γ* = 1. When *T* > *Tu*, the penalty cost was generated, and *γ* > 1. The penalty coefficient was usually a large number [35]. In the formula below, *δ* represents the value inductive coefficient of goods, which indicated how much the consignors cared about the goods value loss while choosing the transportation modes. When *δ*<1, it indicated that the consignors cared more about transportation fee than the goods value loss. When *δ*>1, it indicated that the consignors cared more about the goods value loss than the transportation fee. The penalty coefficient and the inductive coefficient could be calibrated through historical data and statistics.

Thus, the transportation fee *Gi* of transportation mode *i* selected by the consignor was

$$\begin{array}{c} G_{i}=\tau_{i}+\delta \cdot (\gamma ∙\Delta P-B^{p}),\\ =\tau_{i}+\delta \cdot P_{m}[\gamma +e^{-\omega T_{u}}-(\gamma +1)e^{-\omega T_{i}}], \end{array}$$ (5)

where *τ*_*i*_——transportation fee of transportation mode i;

*δ*——goods value inductive coefficient;

*γ*——penalty coefficient;

*P*_*m*_——Original value of goods;

*ω*——Value reduce rate, constant;

*T*_*i*_——The transportation time of transportation mode i;

*T*_*u*_——Expected transportation time.

Other factors such as the customer relationship, satisfaction, and brand effect were expressed by the random term *εi* of the function below. It was assumed that *εi* was independent and it followed the Gumbel distribution (0, *θ*). Then the variance of the utility perceptual error of the transport mode *i* for the cargo owner was [36]

$$\sigma^{2}(\varepsilon_{i})=\frac{\pi^{2}}{6\theta^{2}}.$$ (6)

According to the utility theory and the nature of the Gumbel distribution, the random utility of transport mode *i* was

$$U_{i}={-G}_{i}+\varepsilon_{i}.$$ (7)

Therefore, the probability of being chosen or the market share of transport mode *i* was [37, 38]

$$D_{i}=\frac{exp(-\theta ∙G_{i})}{\sum_{j=1}^{4}exp(-{\theta ∙G}_{j})},$$ (8)

where *θ* is a discrete parameter that was used to measure the utility perception error level of the goods owner [39]. Some research suggested that *θ* was related to the main factors of decision making and it could be estimated by the reciprocal of the absolute value of the main decision quantity. This meant that the decision-making for the goods transportation, transportation cost, and time was the main decision-making factor, so the reciprocal of the absolute value of the generalized cost average was applied to estimate the value of *θ*, which was

$$\theta =\frac{1}{\left|\bar{G_{i}}\right|}.$$ (9)

Therefore, according to Formulas (7) and (8), the expectation of the general cost of the expected transportation time was

$$G_{i}=-\tau_{i}-\delta \cdot P_{m}∙[\gamma +\exp(-\omega T_{u})-(\gamma +1)\exp(-\omega T_{i})].$$ (10)

It was assumed that the transportation method *Tri* applied the service optimizing strategy, which brought the owner additional utility *Si*, and *Si* could be regarded as the service level of transportation method *TRi*. The cost that was required to be achieved at this level was *μiSi2*, and the function of cost was a quadratic function that could satisfy the law of returns increase. This meant that the cost to increase the level of service would increase with the improvement of the service quality, and the higher the quality was, the faster the cost would increase [31, 40, 41].

Then in the evolutionary game, the utility function of the owner was

$$U_{i}^{E}=-G_{i}+S_{i}+\varepsilon_{i}.$$ (11)

The market share of transportation mode *i* in the evolutionary game was

$$D_{i}^{E}=\frac{exp[\theta^{E}∙(-G_{i}+S_{i})]}{\sum_{j=1}^{2}exp(\theta^{E}∙(-G_{j}+S_{j}))}.$$ (12)

The utility perceptual error parameter *θ*^*E*^ in the evolutionary game was

$$\theta^{E}=\frac{1}{\left|\bar{-G_{i}+S_{i}}\right|}.$$ (13)

It was assumed that the total demand of the international cargo transportation market was *Q*. Then the demand for transportation mode *i* was

$$Q_{i}=Q\cdot D_{i}^{E}.$$ (14)

It was assumed that the market price of the transport mode *TRi* was *τi*. Then the profit function of the transport mode *TRi* in the evolutionary game was

$$\Pi_{1}=Q_{1}\cdot (\tau_{1}+A-C_{1})-\mu {S_{1}}^{2},0\leq A\leq p_{1},$$ (15)

$$\Pi_{2}=Q_{2}\cdot (\tau_{2}-C_{2})-\mu {S_{2}}^{2}.$$ (16)

If the chance of CR Express *TR*_1_ to choose a service optimization strategy was *α*, then the chance of not selecting the optimization strategy was 1−*α*. The probability for shipping *TR*_*2*_ to select the service optimizing strategy was *β*. By contrast, the chance of not selecting the optimizing strategy was 1−*β*.

The strategy set of CR Express and shipping was recorded as (*TR*_*1*_, *TR*_*2*_). The superscript “ss” indicates that the strategy set applied (using service optimizing, using service optimizing). The superscript “sn” represents (using service optimizing, not using service optimizing), superscript “ns” means (not using service optimizing, using service optimizing) situations, and “nn” is used to show (not using service optimizing, not using service optimizing) situations. The payment matrix of the evolution game of CR Express and shipping is shown in Table 2 below.

**Table 2:** Payment matrix of evolution game between freight train and shipping.

	Adopt a service optimization strategy (β)	No service optimization strategy (1-β)
Adopt a service optimization strategy (α)	$\Pi_{1}^{ss},\;\Pi_{2}^{ss}$	$\Pi_{1}^{sn},\;\Pi_{2}^{sn}$
No service optimization strategy (1−*α*)	$\Pi_{1}^{ns},\;\Pi_{2}^{ns}$	$\Pi_{1}^{nn},\;\Pi_{2}^{nn}$

For CR Express, when selecting the service optimization strategy, the revenue was

$$E_{1S}=\beta \Pi_{1}^{ss}+(1-\beta )\Pi_{1}^{sn}.$$ (17)

When not choosing the service optimization strategy, the revenue was

$$E_{1N}=\beta \Pi_{1}^{ns}+(1-\beta )\Pi_{1}^{nn}.$$ (18)

Then the expected revenue of CR Express was

$$\begin{array}{c} E_{1}=\alpha E_{1S}+(1-\alpha )E_{1N},\\ =\alpha \beta \Pi_{1}^{ss}+\alpha (1-\beta )\Pi_{1}^{sn}+(1-\alpha )\beta \Pi_{1}^{ns}+(1-\alpha )(1-\beta )\Pi_{1}^{nn}. \end{array}$$ (19)

Thus, the replicator dynamic equation of the service optimization strategy of CR Express selection could be written as

$$\begin{array}{l} \frac{d\alpha }{dt}=\alpha (E_{1S}-E_{1}),\\ \;=\alpha (1-\alpha )\beta \Pi_{1}^{ss}+\alpha (1-\alpha )(1-\beta )\Pi_{1}^{sn}-\alpha \beta (1-\alpha )\Pi_{1}^{ns}-\alpha (1-\alpha )(1-\beta )\Pi_{1}^{nn},\\ \;=\alpha (1-\alpha )[\beta (\Pi_{1}^{ss}+\Pi_{1}^{nn}-\Pi_{1}^{sn}-\Pi_{1}^{ns})+(\Pi_{1}^{sn}-\Pi_{1}^{nn})]. \end{array}$$ (20)

For the maritime carrier, when selecting the service optimization strategy, the revenue was

$$E_{2S}=\alpha \Pi_{2}^{ss}+(1-\alpha )\Pi_{2}^{ns}.$$ (21)

When not choosing the service optimization strategy, the revenue was

$$E_{2N}=\alpha \Pi_{2}^{sn}+(1-\alpha )\Pi_{2}^{nn}.$$ (22)

Then the expected revenue of the maritime carrier was

$$E_{2}=\alpha \beta \Pi_{2}^{ss}+(1-\alpha )\beta \Pi_{2}^{ns}+\alpha (1-\beta )\Pi_{1}^{sn}+(1-\alpha )(1-\beta )\Pi_{2}^{nn}.$$ (23)

Thus, the replicator dynamic equation of the service optimization strategy of maritime carrier selection could be written as

$$\begin{array}{c} \frac{d\beta }{dt}=\alpha \beta (1-\beta )\Pi_{2}^{ss}+\beta (1-\alpha )(1-\beta )\Pi_{2}^{ns}-\alpha \beta (1-\beta )\Pi_{1}^{sn}-\beta (1-\alpha )(1-\beta )\Pi_{2}^{nn},\\ =\beta (1-\beta )[\alpha (\Pi_{2}^{ss}+\Pi_{2}^{nn}-\Pi_{2}^{sn}-\Pi_{2}^{ns})+(\Pi_{2}^{ns}-\Pi_{2}^{nn})]. \end{array}$$ (24)

Hence, $M_{1}=\Pi_{1}^{ss}+\Pi_{1}^{nn}-\Pi_{1}^{sn}-\Pi_{1}^{ns},M_{2}=\Pi_{2}^{ss}+\Pi_{2}^{nn}-\Pi_{2}^{sn}-\Pi_{2}^{ns},N_{1}=\Pi_{1}^{sn}-\Pi_{1}^{nn},N_{2}=\Pi_{2}^{ns}-\Pi_{2}^{nn}$, and after substituting the Formulas (20) and (24), the parallel equations were obtained as shown below:

$$\left\{ \begin{array}{c} \frac{d\alpha }{dt}=\alpha (1-\alpha )(\beta M_{1}+N_{1})=0\\ \frac{d\beta }{dt}=\beta (1-\beta )(\alpha M_{2}+N_{2})=0 \end{array}\right..$$ (25)

The solution system could obtain five local equilibrium points for the dynamic system (25), which were $O_{1}(0,0),O_{2}(0,1),O_{3}(1,0),O_{4}(1,1),O_{5}\left(-\frac{M_{2}}{N_{2}},-\frac{M_{1}}{N_{1}}\right)$

## Market segmentation

There are many kinds of goods transported by the CR express. Research on different types of goods can better provide targeted advice for the sustainable development of CR Express.

To face customer needs transforming from identical to heterogeneous, Smith Wendell initially proposed the concept of market segmentation in 1956. This concept has been the focus of business and academic circles until the present.

Market segmentation refers to dividing the main market into several sub-markets consisting of consumer groups with similar demands according to heterogeneous consumer demands [42].

For the international freight transport market, total supply exceeds demand, while a single transportation mode cannot fully meet all kinds of needs. According to heterogeneous demands from different groups, operators should apply different competitive strategies combined with their own characteristics to finally increase market share.

Based on the difference of goods properties, in this research, HAYASHI’s theory of quantification III was used to classify the railway transportation categories based on the “Classification of Railway Transportation Items”. For different categories of goods, operators could perform different service modes. The goods properties included the (1) goods value, (2) timeliness, (3) container suitability, (4) transport batch, and (5) transport conditions.

Based on theory of quantification III, Matlab was applied for calculation, which led to the 15 characteristic roots corresponding to the cargo response matrix, and the two largest characteristic roots were selected to calculate the corresponding characteristic vectors as b1 and b2. The Hayashi quantitative theory III is a multivariate analysis method based on the construction of a "0–1" attribute judgment matrix and the calculation of a vector value in order to deal with qualitative and quantitative variables. According to b1 and b2, along with Formula (26),

$$Y_{i}=\frac{1}{m+s}(\sum_{j=1}^{m}b_{j}\delta_{i}(j)+\sum_{l=1}^{s}\alpha_{l}U_{il}),i=1,2,\cdots ,n.$$ (26)

Through the above formula, we obtained the first characteristic *Y*_*1*_ and the second characteristic *Y*_*2*_ of 30 categories of goods, which could be used as the projection of the sample vector on the *x* and *y* axes. Obviously, based on the projection results and general understanding, the goods were classified as the following five types, as shown in Table 3 below.

**Table 3:** Classification of railway transportation goods.

Goods types		Threshold value 7.5
Cement	Coal	High-value, low-time-sensitive goods
Steel and nonferrous metals	Oil	
Wood	Coke	
Cereals	Metal ore	
Cotton	Nonmetallic ore	
Salt	Phosphate rock	
Agricultural machinery	Mineral building materials	
Industrial machinery		
Agricultural and sideline products	Semiconductor material	High-value, low-time-sensitive goods
Paper and stationery	Glass, glass fiber, and their products	
Furniture, luggage, daily groceries	Chemical fertilizers and pesticides	
Arts and crafts, exhibits	Chemicals	
	Metalware	
Textiles, leather, fur, and its product	Medical instruments, instruments, scales, measuring instruments	High-value and high-time-sensitivity goods
Drink, food, and tobacco products	Electronics, electrical machinery	
Pharmaceuticals	Fresh goods	Low-value and high-time-sensitivity goods

1. High-value and high-time-sensitivity goods. The value of these goods decreased rapidly over time, which meant that higher freight was acceptable for consignors with the transportation demand for this type of goods.
2. High-value, low-time-sensitive goods. The value of these goods changes little over time, so even though their value was high, the freight was expected to be as low as possible.
3. Low-value and low-time-sensitive goods. The value of the goods was low and it changed little over time, so the first priority of a consignor was the low freight.
4. Low-value and high-time-sensitivity goods. The value of these goods was low, while the value changed by a large amount over time. The shorter transportation time was the key factor for these goods.
5. Other goods that did not belong to the above four categories had certain characteristics, such as being inflammable, explosive, and radioactive goods.

### Case study

CR Express (Chongqing-Duisburg) departed from Chongqing, through Xi’an, Lanzhou, Urumqi, Alataw Pass, Kazakhstan, Russia, Belarus, Poland, to Duisburg in Germany, with a total length of 11179 km, as shown in Table 4.

**Table 4:** The CR Express (Chongqing-Duisburg) sections of the transport distance.

Transit sections	Transit countries	Transport distance (km)
Chongqing-Alataw Pass	China	3777
Alataw Pass/Dostyk-Ilizburg	Kazakhstan	3412
Ilizburg-Krasnoe	Russia	1497
Krasnoye-Brest	Belarus	587
Brest-Malaszewicze-Duisburg	Warsaw, Germany (EU)	1906

From Eq (1), the unit cost $c_{1}^{op}$ of the carriers of CR Express was

$$\begin{array}{l} C_{1}=c_{1}^{\mathrm{in}}\cdot d_{1}^{\mathrm{in}}+c_{1}^{\mathrm{out}}\cdot d_{1}^{\mathrm{out}}+c_{1}^{\mathrm{ex}},\\ \;=3777\times 0.5+3412\times 0.7+1497\times 0.55+587\times 0.7+1906\times 1+155+111,\\ \;=7683.15\;\$/FEU. \end{array}$$ (27)

From known literature and resources, the current market price of CR Express (Chongqing-Duisburg) at the time of this study was 6000 USD/FEU, which was much lower than the cost of railway transportation. This meant that the main method for CR Express to make profits was the financial subsidies, and it was assumed that the average amount of a subsidy was A = 2000 USD/FEU. Once the financial subsidies were withdrawn, the price of CR Express would increase. This would also influence the market competitiveness and strategic choice of CR Express operators, so the different price levels were also discussed, as described in the following sections. The prices of CR Express are presented in table below. The price of transportation without a subsidy was a reasonable assumption that was based on transportation unit cost.

According to literature [43], the average shipping cost of ocean routes between China to Germany was C2 = 3000 USD/FEU.

It was assumed that the expected transporting time was relatively long, which meant that both the transportation time of CR Express and shipping would be within the expected time without any value loss of carried goods.

The simulation data used for the model according to the survey data is presented in Table 5, below. During the analysis of evolution game simulation, the different initial probability combinations were selected.

**Table 5:** Data for evolutionary game model.

Parameter	Specification	Value
$\tau_{1}^{\left(0\right)}$	The current market price of China-Europe freight trains	$6000 / FEU
$\tau_{1}^{\left(1\right)}$	Subsidy after withdrawal of transport prices	$8000 / FEU
*τ*_2_	Sea present market price	$8000 / FEU
$P_{m}^{\left(1\right)}$	Laptop (high value high time sensitive cargo) initial value	1.47 million dollars
$P_{m}^{\left(2\right)}$	Auto parts (high value low time sensitive goods) initial value	1 million dollars
$P_{m}^{\left(3\right)}$	Fruit (low value high time sensitive goods) initial value	500000 dollars
$P_{m}^{\left(4\right)}$	Industrial accessories (low value low time sensitive goods) initial value	100000 dollars
*T*_*u*_	Expected shipping time period	50 days
*δ*	Cargo value inductance	0.5
*ω* ^(1)^	Laptop (high value high time sensitive goods) depreciation rate	0.0433%
*ω* ^(2)^	Auto parts (high value low time sensitive goods)	0.0012%
*ω* ^(3)^	Fruit (low value high time sensitive goods) derogatory rate	0.1%
*ω* ^(4)^	Industrial accessories (low value low time sensitive goods) derogatory rate	0.001%
*A*	The amount of government subsidies for CR Express	2000 dollars
*C*_1_	Unit transportation cost of CR Express	$7683.15 / FEU
*C*_2_	Unit shipping cost	$3000 / FEU
*S*_*i*_	I = 1,2, service level	2000
*μ*	I = 1,2, service input cost coefficient	0.01

The government’s subsidies for China-Europe Express trains played an important role in the price competition between CR Express delivery and shipping. In the future, with the gradual withdrawal of government subsidies for CR Express, the price gap between CR Express and shipping will become smaller, and their competition in the freight market will become more intense. Therefore, as described in the following discussion, the situations were divided into impact with subsidy and impact without subsidy to analyze the evolution of the game separately.

### (1) Impact with subsidy

The market share and profit of CR Express and shipping in different situations is shown in Table 6. In this table, (s/n, s/n) stands for different strategies applied by CR Express and the maritime transportation, where “s” indicates the use of the service optimizing strategy and “n” indicates not using the service optimizing strategy.

**Table 6:** Market share and profit (with subsidies) of CR Express and shipping for different service levels.

Cargo type	Data type	(*s*,*s*)	(*s*,*n*)	(*n*,*s*)	(*n*,*n*)
Laptop (high value high time sensitive goods)	*π*_1_ (×10^7^dollar)	14.6460	14.5670	13.7590	13.7840
	*π*_2_ (×10^7^dollar)	1.8778	2.0072	3.2831	3.2486
	*d*_1_	92.47%	91.97%	86.85%	87.01%
	*d*_2_	7.53%	8.03%	13.15%	12.99%
Auto parts (high value low time sensitive goods)	*π*_1_ (×10^7^dollar)	5.0296	7.7594	3.9920	6.1802
	*π*_2_ (×10^7^dollar)	17.0530	12.7490	18.6790	15.2470
	*d*_1_	31.77%	49.00%	25.20%	39.01%
	*d*_2_	68.23%	51.00%	74.80%	60.99%
Fruit (low value high time sensitive goods)	*π*_1_ (×10^7^dollar)	14.0990	14.0770	12.8700	13.0380
	*π*_2_ (×10^7^dollar)	2.7411	2.7800	4.6862	4.4256
	*d*_1_	89.02%	88.88%	81.24%	82.30%
	*d*_2_	10.98%	11.12%	18.76%	17.70%
Industrial accessories (low value high time sensitive goods)	*π*_1_ (×10^7^dollar)	4.5859	7.4212	3.6939	5.9084
	*π*_2_ (×10^7^dollar)	17.7520	13.2830	19.1670	15.6760
	*d*_1_	28.97%	46.87%	23.32%	37.29%
	*d*_2_	71.03%	53.13%	76.88%	62.71%

As Table 6 indicates, for auto parts, after improving the service level, the market share of CR Express increased from 39.01% to 49.00%. For fruits, when CR Express did not use the service optimizing strategy, even maritime operators applied the service optimizing strategy, and the market share of the maritime share increased from 17.70% to 18.76%. For industrial accessories, after improving the service level, the market share of CR Express increased from 37.29% to 46.87%. When both modes were in the same high service level, the difference of the market share was higher than the difference of the low service level. This indicated that improving service was helpful to expanding the competitive advantage.

For this model, the evolution time *t* was set from 0 to 100 and the evolution processes of various goods were obtained for different initial selections. The following selection of initial probability combinations (α, β) = (0.2, 0.2), (0.2, 0.8), (0.8, 0.2), (0.8, 0.8) reflected the evolution situations of CR Express and maritime transportation.

**Fig 3:**
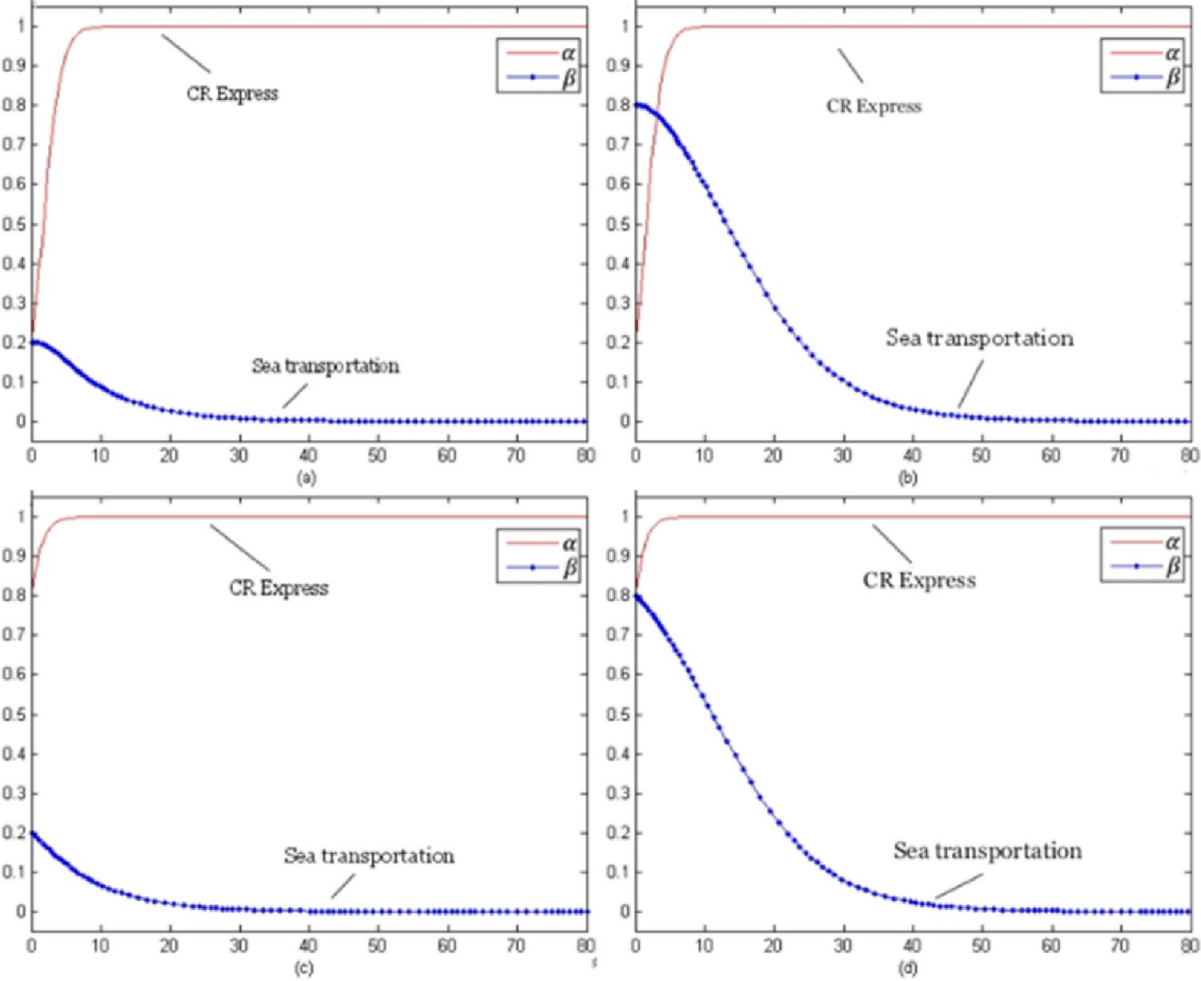
Evolution trends of laptop transportation competition game (with subsidies).

Fig 3 presents the evolutionary game trend of competition of laptop transportation. For different initial probabilities, the evolutionary stable strategy combinations were (using service optimization strategy, no service optimization strategy) shown as (s, n).

**Fig 4:**
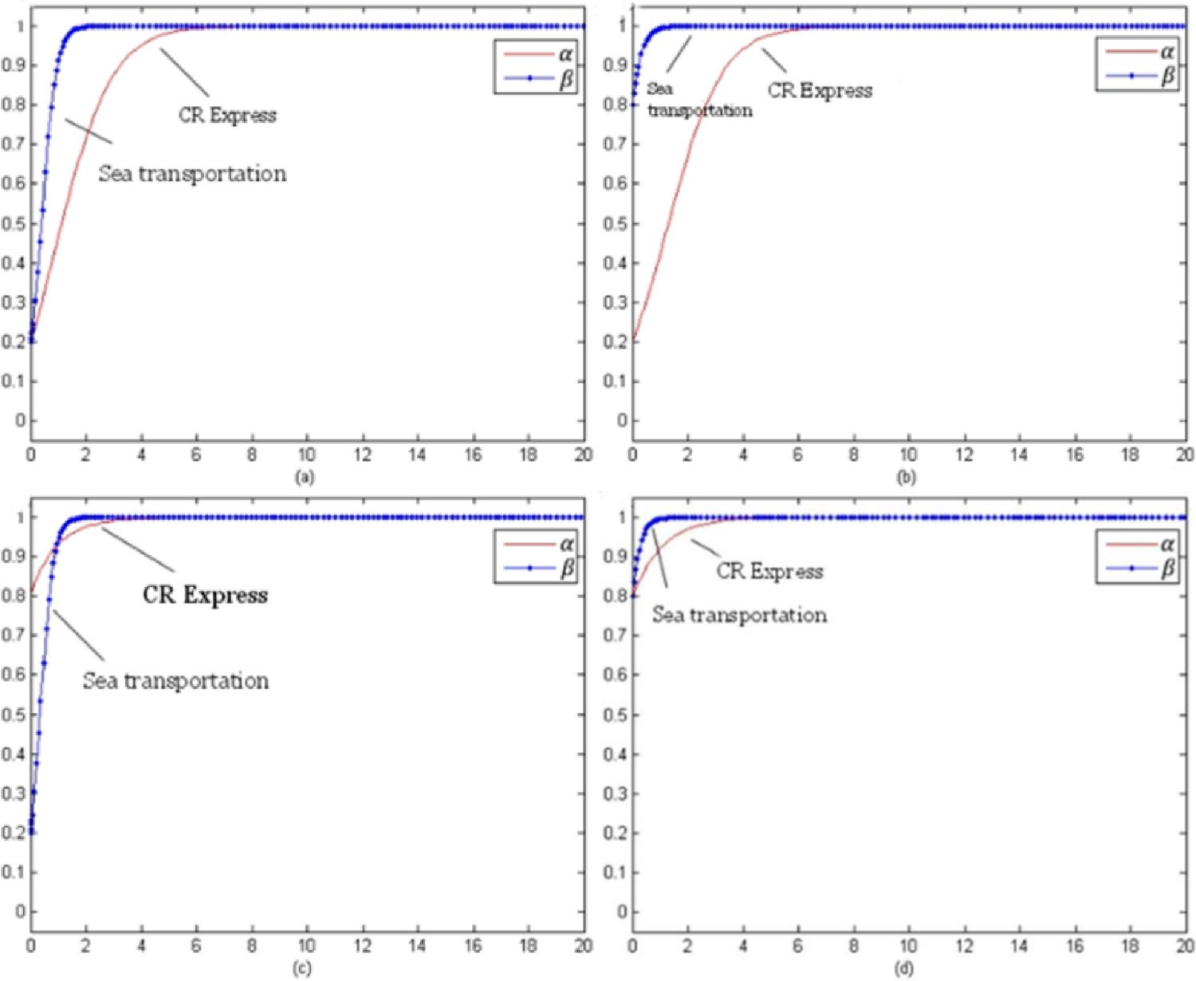
Game evolution trend of auto parts transportation competition (with subsidies).

Fig 4 shows the evolutionary trend of the competition game of auto parts transportation. For different initial selection probabilities, the evolutionary stability strategy was (using the service optimization strategy, using the service optimization strategy), which could be presented as (s, s).

**Fig 5:**
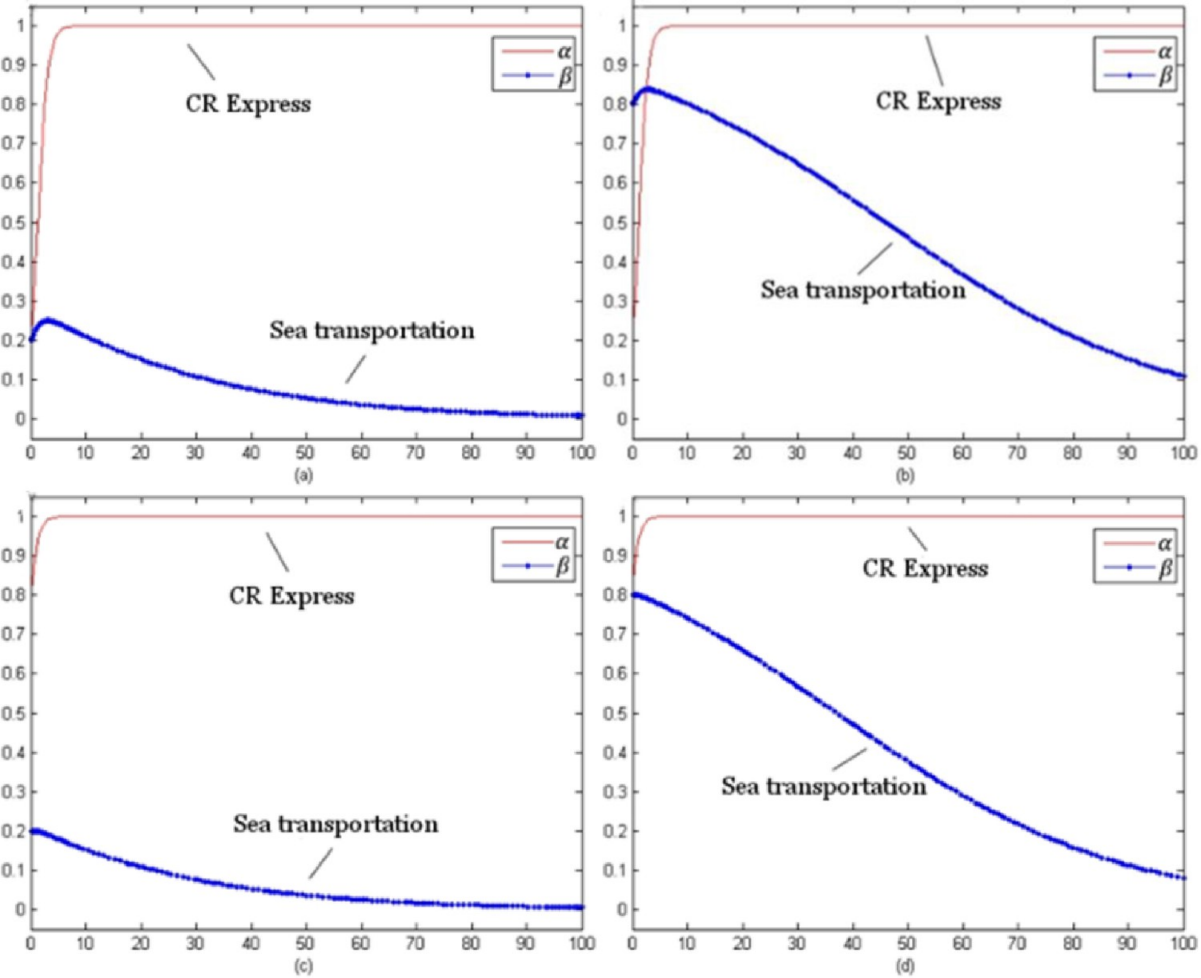
Game evolution trend of fruit transportation competition (with subsidies).

Fig 5 indicates the evolutionary trend of the fruit transportation. For different initial selection probabilities, the evolutionary stability strategy was (using the service optimization strategy, not using the service optimization strategy) which could be presented as (s, n).

**Fig 6:**
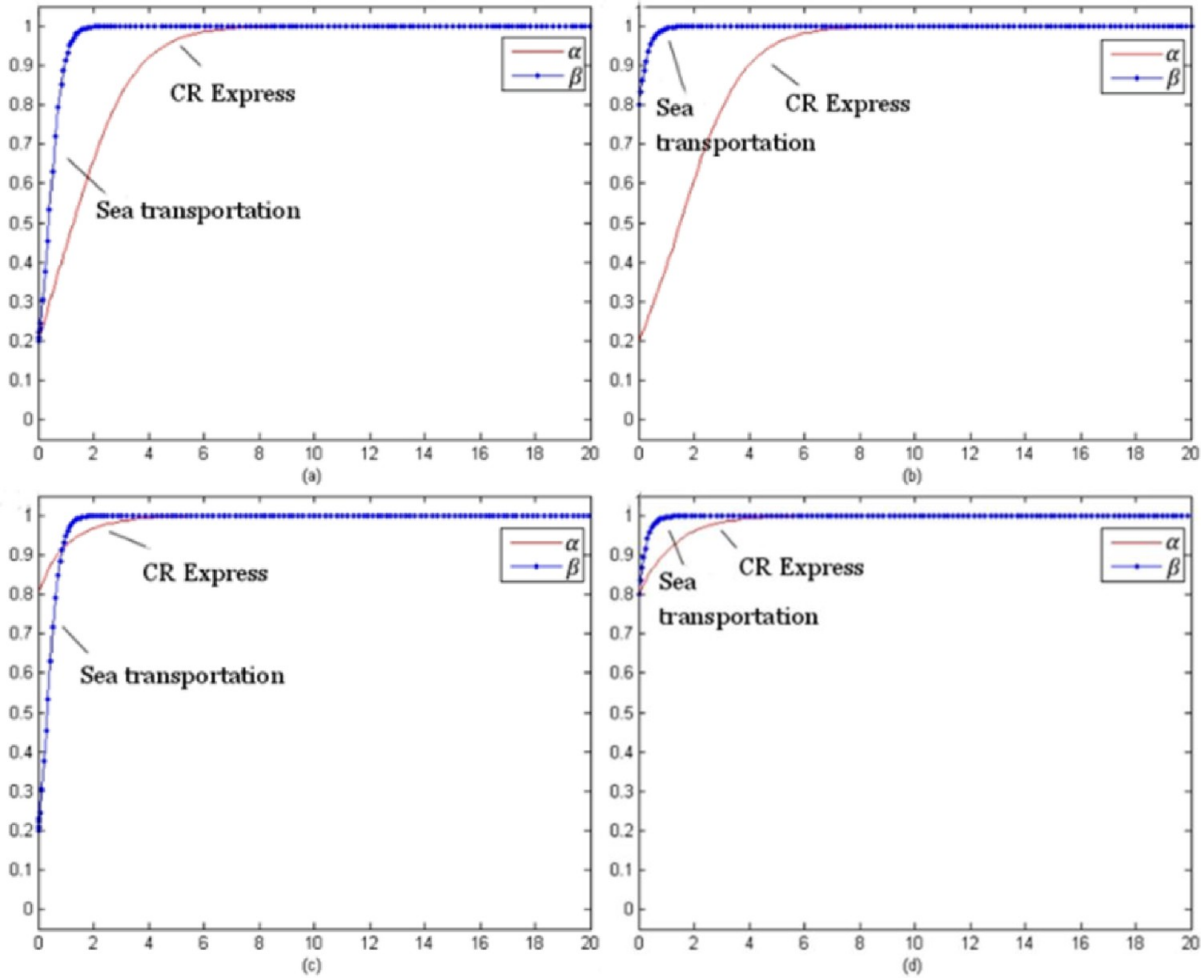
Evolution trend of competition game of industrial accessories transportation (with subsidies).

Fig 6 above presents the evolution trend of the industrial parts transportation. With different initial selection probabilities, the evolutionary stability strategy combination of CR Express and maritime transportation was (using service optimization strategy, using service optimization strategy) which could be referred to as (s, s).

### (2) No subsidy impact

Based on same logic, according to the model, the profits and market share of CR Express and shipping without the effect of the subsidy is shown in Table 7.

**Table 7:** Market share and profit (without subsidy) of CR Express and shipping at different service levels.

Cargo type	Data type	(*s*,*s*)	(*s*,*n*)	(*n*,*s*)	(*n*,*n*)
Laptop (high value high time sensitive goods)	*π*_1_ (×10^7^dollar)	13.7550	13.7800	12.7520	12.9060
	*π*_2_ (×10^7^dollar)	3.2831	3.2486	4.8722	4.6342
	*d*_1_	86.85%	87.01%	80.50%	81.46%
	*d*_2_	13.15%	12.99%	19.50%	18.54%
Auto parts (high value low time sensitive goods)	*π*_1_ (×10^7^dollar)	3.9880	6.1762	3.4535	5.2080
	*π*_2_ (×10^7^dollar)	18.6970	15.2470	19.5460	16.7820
	*d*_1_	25.20%	39.01%	21.80%	32.87%
	*d*_2_	74.80%	60.99%	78.20%	67.13%
Fruit (low value high time sensitive goods)	*π*_1_ (×10^7^dollar)	12.8660	13.0340	11.5980	11.9500
	*π*_2_ (×10^7^dollar)	4.6862	4.4256	6.6942	6.1418
	*d*_1_	81.24%	82.30%	73.21%	75.43%
	*d*_2_	18.76%	17.70%	26.79%	24.57%
Industrial accessories (low value high time sensitive goods)	*π*_1_ (×10^7^dollar)	3.6899	5.9044	3.2338	4.9905
	*π*_2_ (×10^7^dollar)	19.1670	15.6760	19.8930	17.1250
	*d*_1_	23.32%	37.29%	20.41%	31.50%
	*d*_2_	76.68%	62.71%	79.59%	68.50%

By setting the model evolution time t from 0 to 100, the evolution process of each type of cargo under different initial selection probabilities could be obtained. The following selection of initial probability combinations was (α, β) = (0.2, 0.2), (0.2, 0.8), (0.8, 0.2), (0.8, 0.8), which reflected the stable evolution of the CR Express and shipping for different combinations of probabilities.

**Fig 7:**
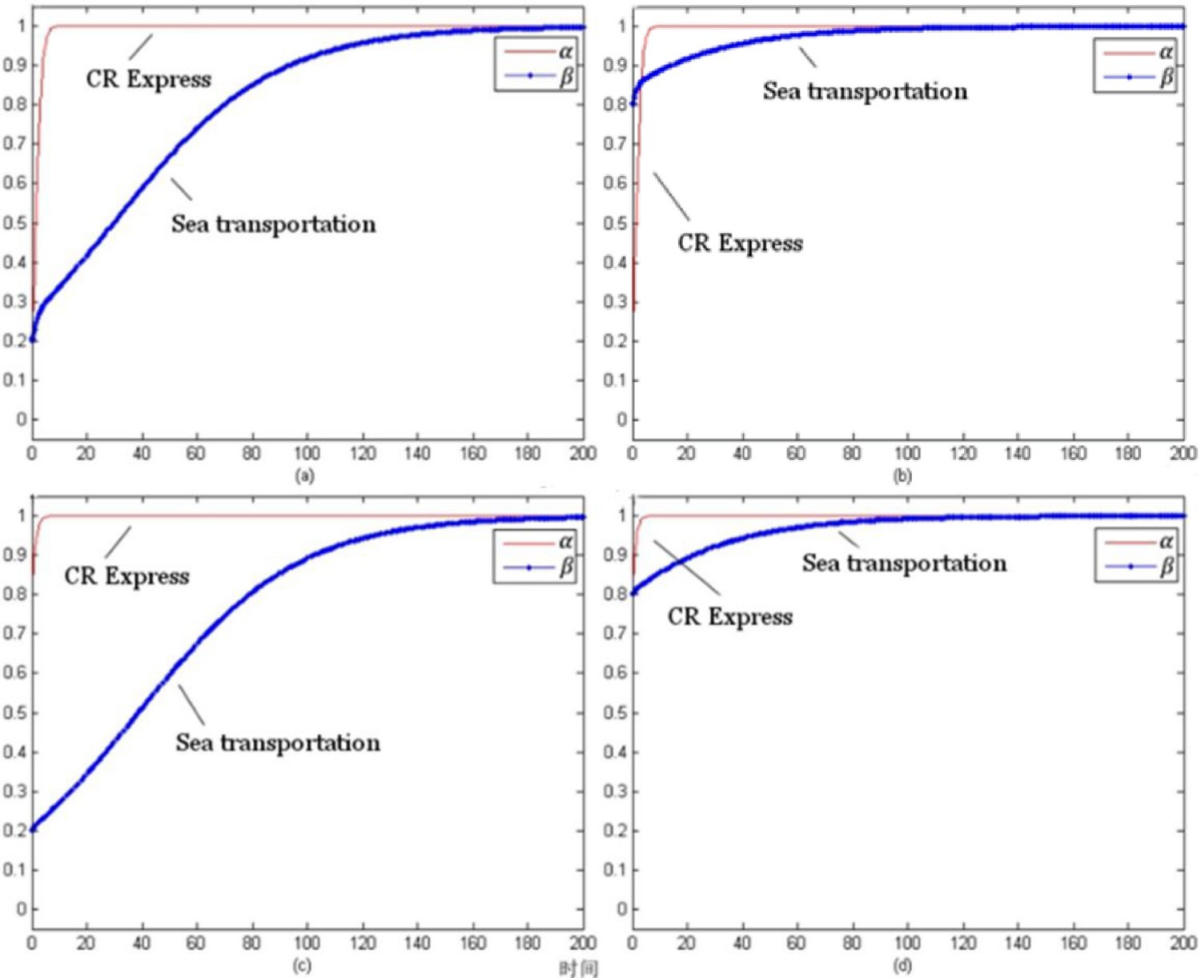
Evolution trend of laptop transportation competition game (without subsidy).

Fig 7 evolution trend of laptop transportation competition game (without subsidy) presents the evolutionary trend of laptop transportation competition without subsidies. In contrast to the evolutionary trend with subsidies shown in Fig 3, for different initial selection probabilities, the evolutionary stable strategy set was (adopt a service optimization strategy, adopt a service optimization strategy), which could be presented as (s, s).

**Fig 8:**
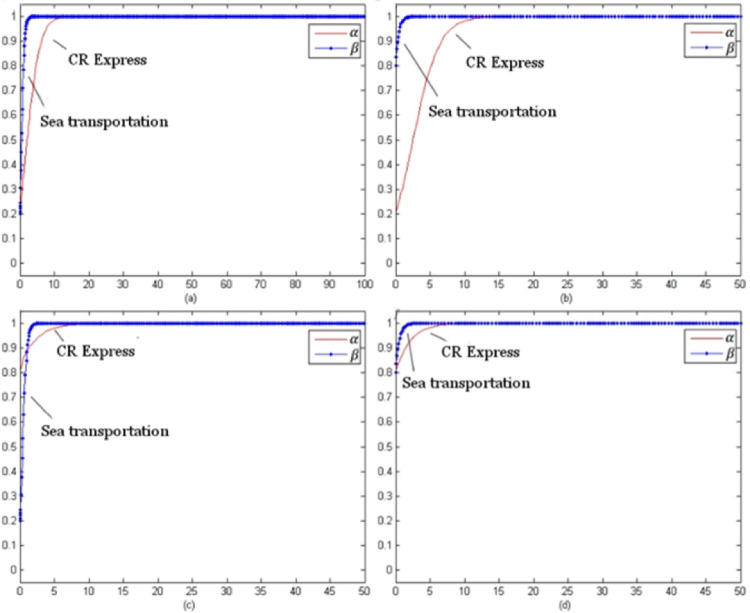
Game evolution trend of auto parts transportation competition (without subsidies).

Fig 8 presents the non-subsidy situation and the evolution trend of the auto parts transportation competition game, which was similar to the evolution trend of the subsidized trend shown in Fig 4. The evolutionary stability strategy combination was (using the service optimization strategy, using the service optimization strategy), which could be shown as (s, s).

**Fig 9:**
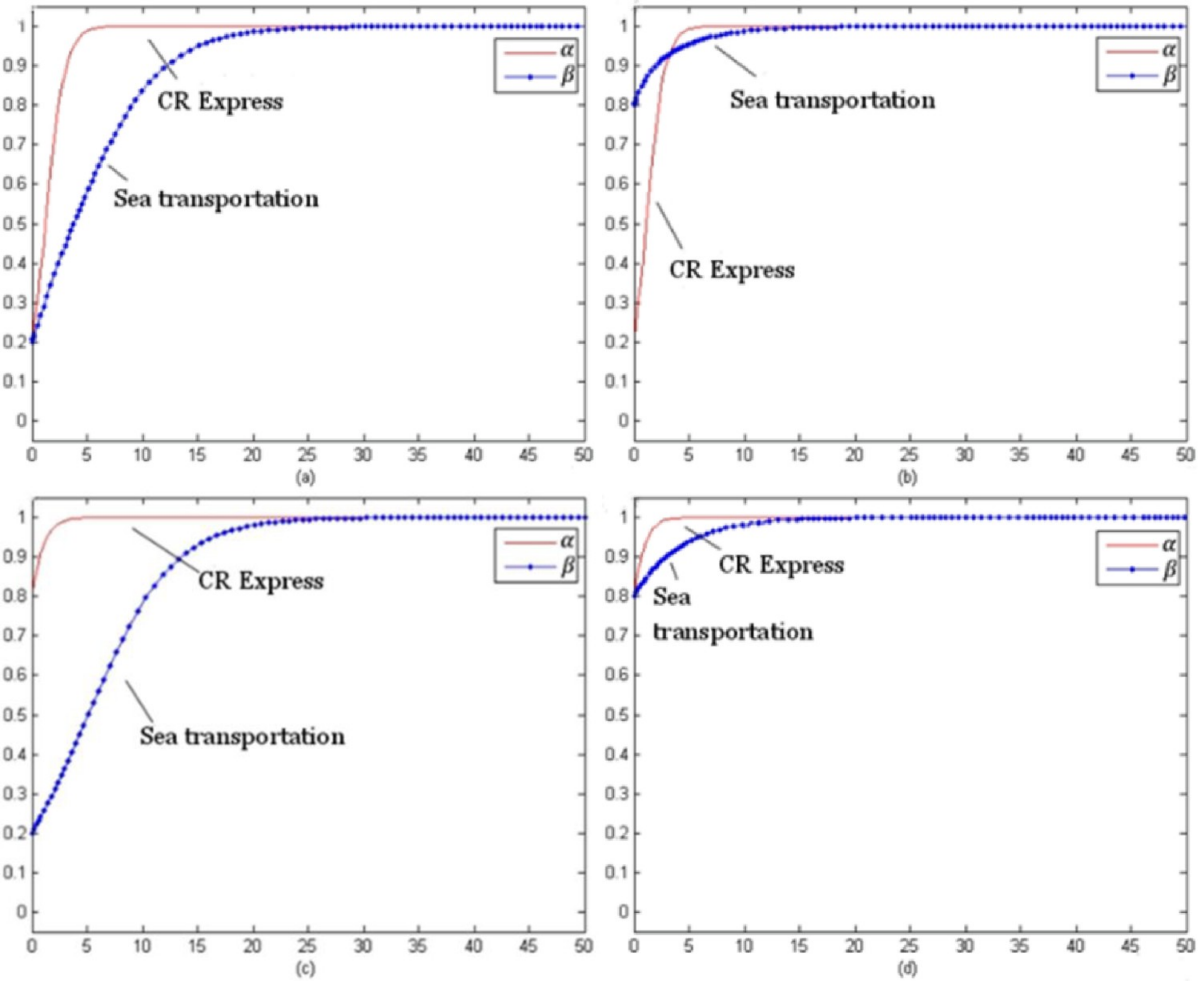
Game evolution trend of fruit transportation competition (without subsidies).

Fig 9 shows the evolutionary trend of the fruit transportation competition game for the non-subsidies situation. Unlike the evolution trend with the subsidy, the evolutionary stability strategy combination was (using the service optimization strategy, using the service optimization strategy), which was presented as (s, s).

**Fig 10:**
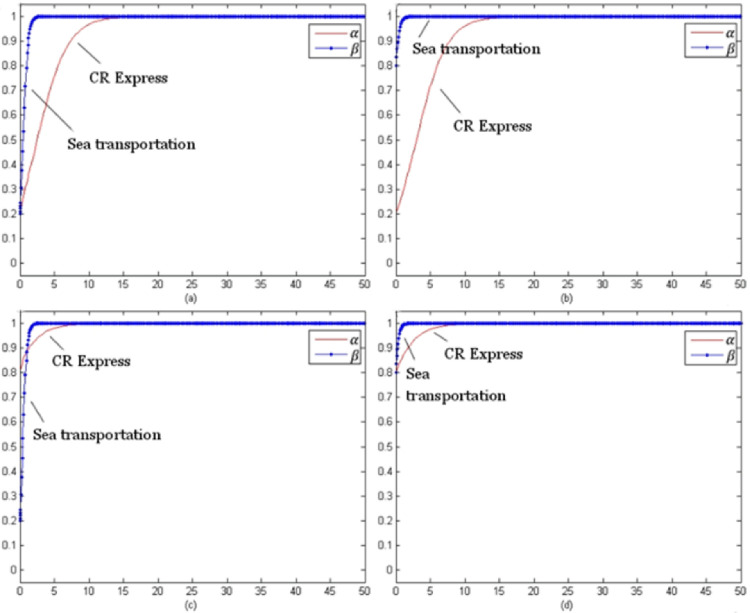
Evolution trend of competition game of industrial accessories transportation (without subsidies).

Fig 10 presents the evolution trend of the competition game for industrial parts transportation without subsidies. The trend was roughly the same as the trend with subsidies shown in Fig 6, and the evolutionary stability strategy was (using the service optimization strategy, using a service optimization strategy), which was (s, s).

## Result

For the transport of high-value and time-sensitive goods such as laptops, CR Express (without subsidies) had a larger competitive advantage in the market based on its shorter transportation time. With a high service level, CR Express attracted more freight demands. For high-value and low-time-sensitive goods like auto parts, based on the lower prices, maritime transportation maintained its traditional advantage. For this situation, CR Express had to improve its service to enhance competitiveness to gain a larger market share.

By comparing the data of Tables 6 and 7, it could be seen that due to the withdrawal of subsidies, the price of CR Express increased, and the trend of the market share started to decrease, but there was not a large drop. This indicated that even without the supply of governments, CR Express was still competitive, especially for the international cargo transportation of high-time-sensitiveness goods such as fruits and laptops.

As shown in Fig 3, as the evolutionary stable strategy combinations indicated, the service optimization strategy was the optimal strategy for CR Express. Additionally for maritime transportation, the profits for the situation of not using service level improvement were greater than those for improving the service level were. Therefore, even if the probability of selecting the service optimization strategy was high in the initial stage, the probability would keep decreasing with time and finally stabilize at zero. For the high-value and high-time-sensitiveness goods, the transportation time of CR Express was much shorter than that of maritime transportation, so the potential market competitiveness of CR Express was much higher than that of maritime transportation. Therefore, for the long-term version, CR Express needed to improve service level, while maritime transportation would not improve service positively, and it would face the competition negatively.

As shown in Fig 4, as the evolutionary stable strategy combinations indicated, for both CR Express and maritime transportation, applying the service optimization strategy was the optimal strategy. This was because the higher freight rate was acceptable for high-value and low-time-sensitiveness goods based on the higher value of the goods. Therefore, even though maritime transportation provided an advantageous price, the difference between CR Express and shipping was acceptable. When CR Express applied the service optimizing strategy, the competition gap with CR Express would be further narrowed, which would make maritime transportation operators improve the service level to maintain their competitive advantages.

As shown in Fig 5, as the evolutionary stable strategy combinations indicated, for CR Express, the service optimization strategy was the best strategy. Since both fruits and laptops were high-time-sensitiveness goods, CR Express maintained its higher competitive advantages, so its evolution trend was similar to the trend shown in Fig 3. The difference was that in the early stage of evolution, the selection probability of maritime transportation increased a little and then decreased and finally stabilized at zero when the selection probability of CR Express was low. In addition, for fruit transportation, compared to laptop transportation, the probability of choosing the service optimization strategy for maritime transportation declined more slowly. This was because maritime transportation could satisfy the transportation demands with lower prices, so it was more attractive for low-value goods like fruits. Additionally, for high-time-sensitiveness goods like laptops, the goods value was much higher, so the acceptable price would be higher. That was the reason why the market share difference between CR Express and maritime transportation for the fruits transportation market was lower than that of the laptops transportation market share. In the long term, shipping would not adopt service optimization strategies and face competition in a negative way, while CR Express could obtain greater market share through improving service levels.

As shown in Fig 6, since both industrial parts and auto parts were low-time-sensitive goods, the competitive advantage of maritime transportation was greater than that of CR Express due to the lower freight rates, which caused the evolution trend shown in Fig 6 to be similar to that in Fig 4. Since the value of auto parts was higher and consignors could accept a high transportation price, the competition difference between CR Express and maritime transportation for auto parts transportation was lower than that of industrial parts transportation. Therefore, in the market game, for the transportation similar types of products for CR Express, the strategy adopted could be similar to that of high-value and low-time-sensitive goods, which could effectively improve competitiveness through service optimization.

Fig 7 shows that as the price of CR Express (without subsidies) increased and the difference of the market share between CR Express and shipping decreased, the carriers of shipping trended towards applying a more positive competition strategy. Therefore, the probability of choosing service optimization increased and finally became stable for the service optimization strategy. The probability rise of shipping was slower than that of CR Express. Based on the subsidized and unsubsidized situation analyses, the change of the evolutionary strategy of the laptop transportation game indicated that the government subsidy policy caused significant disruption to the market behavior of CR Express. After the withdrawal of the subsidy, the market competitive advantages of CR Express decreased further, and the market competition between both sides was more intense.

## Conclusions

In the context of the Belt and Road Initiative, CR Express has attracted funds and transportation demands by providing a new transportation mode from China to Europe. The rapid growth of CR Express over the past decade is largely due to the subsidies from the Chinese government. In the future, with the gradual reduction of subsidies for CR Express, the competition between CR Express and maritime transportation will be more intense. This article established a competitive game model to determine the competitive strategies of CR Express. Game participants in this game consisted of CR Express operators, the government, other transportation modes, and consignors. In terms of the competitive strategies, the main types were price strategy, cost strategy, and differentiation strategy. For transportation, the adopted strategies were a price strategy and a differentiation strategy. This research emphasized the differentiation strategy, which was to improve service quality and optimize service. Based on different types of goods, different simulations revealed competitive strategies for both CR Express and maritime transportation.

The result of this research shows that when there is subsidy for CR Express, for high time sensitive goods (including laptop and fruit), the evolutionary stable strategy of maritime transportation is not to use the service optimization strategy, and for low time sensitive goods (including industrial accessories and auto parts), maritime transportation tend to take more active competition strategy, as its evolutionary stable strategy is to use the service optimization strategy. When the subsidy is removed, the price of CR Express trains rose, which led to a corresponding decline in market share for the trains. In the competition between CR Express and maritime transport for highly time-sensitive cargo transportation, the gap between the market share of China–Europe Express and maritime transport narrowed. Therefore, maritime transport would adopt a relatively active competition strategy to improve the service level to compete with CR Express to compete with the market share; and in the competition between CR Express and the maritime transport of low-time-sensitive goods, because maritime transport still had a competitive advantage, the strategy adopted by maritime transport was to improve service. At the same time, for China–Europe Express, whether there were subsidies or not, active competition strategies needed to be adopted to improve service levels. Therefore, this research revealed some market development thoughts for CR Express. Since the provided government subsidies gave CR Express a way to decrease its cost, using a service optimizing strategy was the best strategy for CR Express to increase its market share and achieve quick development.

Furthermore, with this research, it could also be seen that government subsidies greatly interfered with the competition between CR Express and maritime transport. When government subsidies were gradually withdrawn, the competition between CR Express and maritime transport would intensify. Both CR Express and maritime transport would tend to adopt an active competitive strategy that was a service optimization strategy. Therefore, determining how to better coordinate the competitive relationship between CR Express and maritime transport would also be an important research direction in the future.

## Supporting information

S1 Data (XLSX)
